# Patient Satisfaction with Nursing Care in Five Major Departments in a Tertiary Care Centre

**DOI:** 10.31729/jnma.4643

**Published:** 2019-10-31

**Authors:** Sajan Acharya, Calvin Ghimire, Akriti Shrestha, Ashok Kumar Yadav, Seema Bhandari

**Affiliations:** 1 Patan Academy of Health Sciences, Lagankhel, Lalitpur, Nepal

**Keywords:** *nursing care*, *patient satisfaction*, *quality of health care*

## Abstract

**Introduction:**

Patient satisfaction is an important component of quality nursing care and is often determined by the nursing care in any health institution. The aim of the study is to find the presence of satisfaction among in-ward patients of five major wards at a tertiary care hospital regarding the quality of care provided by nursing staff.

**Methods:**

A descriptive cross-sectional study was conducted among 105 patients of Patan Hospital from 3rd July to 3rd August, 2015 after obtaining ethical clearance from Institutional Review Committee. Sample size was calculated and stratified random sampling was done. Data was collected in Microsoft Excel and analyzed in Sta 13.0. Point estimate at 95% Confidence Interval was calculated and frequency and percentage was calculated for binary data. Subgroup analysis was done on the basis of demographic variables.

**Results:**

Among 105 patients, 99 (94.3%) [94.93-95.07 at 95%CI] were satisfied with the nursing care provided at a tertiary care center in Nepal. Among them, 60 (60.6%) were females and 39 (39.4%) were males. Age of the patients ranged from 1 year to 85 years. The length of the stay in the hospital ranged from 2 to 17 days (mean = 5.6 days).

**Conclusions:**

Most of the patients were satisfied with the nursing care provided in a tertiary care centre. Routine nursing care surveys and immediate feedbacks would keep the authorities updated and deliver good health care.

## INTRODUCTION

A major outcome of the quality of health care is patient satisfaction. Patient satisfaction is critical to how well patients do; research has identified a clear link between patient outcomes and patient satisfaction scores.^1^ It is an important component of quality nursing care and is often determined by the nursing care in any health institution.^1,2^ Three major domains that influence the perception of the quality of care are commonly agreed upon to be communication, availability and technical competency.

Patient satisfaction surveys are often used to understand patients’ concerns and determine areas for improvement, including improving communication between physicians and patients. Survey results document progress and allow physicians and staff to maintain high standards.^3^ A study showed that improvements are more likely to occur if staff receives immediate feedback.^4^

The aim of the study is to find the level of satisfaction among in patients of five major wards at a tertiary care hospital regarding the quality of care provided by nursing staff.

## METHODS

A descriptive cross-sectional study was conducted among 110 patients in Patan Hospital, Lalitpur, Nepal after obtaining ethical clearance from Institutional Review Committee. The study was conducted from 3rd July to 3rd August, 2015. Patients from five major wards; surgery, medicine, pediatrics, orthopedics and gynecology/maternity were included in the study. Patients who did not give consent, who had stayed in the ward for <24 hours and who were mentally challenged and unconscious were excluded.

Sample size was calculated using the following formula,

n = Z^2^ × p × q/e^2^

   = 1,96^2^ × 0.5 × 0.5/0.1^2^

   = 96

where,

n = sample size
p = prevalence, 50%
q = 1-p
e = margin of error, 10%
Z = 1.96 at 95% CI

Sample size calculated was 97. Taking non-response rate of 7%, the final sample size was 105. A questionnaire consisting of 3 major subheadings; communication, availability and technical competency, with 5 questions each were provided to the patients.

Stratified random sampling was done. Data was collected in MS Excel and analyzed in SPSS. Point estimate at 95% Confidence Interval was calculated and frequency and percentage was calculated for binary data. Subgroup analysis was done on the basis of age, sex, ethnicity, education, marital status, occupation and admission.

## RESULTS

Among 105 patients, 99 (95%) were satisfied with the nursing care provided in a tertiary care centre. Among them, 60 (60.6%) were females and 39 (39.4%) were males. Age of the patients ranged from 1 year to 85 years. The length of the stay in the hospital ranged from 2 to 17 days (mean = 5.6 days). The majority of participants were Hindu 93 (88.57%). Seventy three (69.52%) had completed their primary level education.

The three domains were assessed in regard to patient satisfaction with the level of nursing care (Table 1).

**Table 1:** Patient satisfaction level.

Patient satisfaction factors	*Score range	Mean	SD
A: Overall	1-4	3.02	0.09
A1: Availability	1-4	2.96	0.65
A2: Communication	1-4	3.12	0.60
A3: Technical Competency	1-4	3.00	0.71

*Patient satisfaction level: 1= Completely dissatisfied – 4=Completely satisfied.

Overall patient’s satisfaction level in regards to communication skills was found to be good (Table 2).

**Table 2:** Level of satisfaction in regards to different sub domains of communication.

Sub-domains	*Range	Mean	SD
Addressing patients	1-4	3.17	0.62
Body language	1-4	3.05	0.57
Patient’s care information	1-4	3.11	0.66
Answers to queries	1-4	3.19	0.56
Listening skills	1-4	3.08	0.59

*Patient satisfaction level: 1= Completely dissatisfied – 4=Completely satisfied.

The overall assessment of availability yielded that most of the patient’s perceived availability of the number of nurses was adequate (Table 3).

**Table 3:** Level of satisfaction in regards to different sub-domains of availability.

Sub-domains	*Range	Mean	SD
No. of day-duty nurses	1-4	2.96	0.61
Time by day-duty nurses	1-4	2.95	0.60
No. of night-duty nurses	1-4	2.94	0.67
Time by night-duty nurses	1-4	2.94	0.66
Time to your queries	1-4	3.02	0.72

*Patient satisfaction level: 1= Completely dissatisfied – 4=Completely satisfied.

The overall assessment of technical competences yielded that most of the patients perceived technical competencies of nurses was adequate (Table 4).

**Table 4:** Level of satisfaction in regards of different sub domains of technical competences.

Sub-domains	*Range	Mean	SD
Skills	1-4	3.05	0.67
Decision making capacity	1-4	2.91	0.73
Explanation about health problems	1-4	3.06	0.63
Explanation about treatment	1-4	3.08	0.67
Help in maintaining hygiene	1-4	2.90	0.83

*Patient satisfaction level: 1= Completely dissatisfied – 4=Completely satisfied.

## DISCUSSION

In our study, 95% were satisfied with the nursing care provided in a tertiary care centre in Nepal. The findings were similar to some national and international studies. Laurent et al. 2006 conducted a study in a tertiary teaching hospital in France aiming to assess the opinions of clinical staff towards the effect of in-patient satisfaction surveys on the quality improvement process. A favorable result of 94% revealed that the patient was able to judge hospital service quality, especially in its relational, organizational and environmental dimensions.^5^

In a study conducted by Gupta et al., overall nursing care was perceived positively by 91% of respondents and recommended that all nurses and hospital administrators need to be more aware of the patient’s views in terms of adequate explanation of the procedure, maintaining privacy and ward environment.^6^ Another study carried out in hospitals of Bhaktapur concluded 64% had positive comments on overall aspect of nursing care.^7^ Subedi D et al. concluded in a study conducted in the Institute of Medicine, Nepal that patients were highly satisfied with the technical competencies of doctors and nurses.^8^

Socio-demographic characteristics were concluded by some studies to be at best a minor predictor of satisfaction.^8^ Patient demographics such as age, gender, income, socioeconomic and general health status impact patients’ responses.^9^ Beyond these obvious characteristics, many other factors such as primary language, parental status, sexual orientation, values, beliefs, or communication style may be associated. The most consistent determinant of patient satisfaction from health care was found to be patient age in various research.

A body of evidence from different countries suggests that older people tend to be more satisfied with health care than do younger people. Regarding gender, some studies show men are more satisfied than women while other studies show contradictory results. A higher level of education was less satisfied with healthcare.^10^ Patients who are more satisfied with their care are more likely to follow medically prescribed regimens and thus contributing to the positive influence on health.^11^

The limitation of our study is that it is conducted in small settings so results cannot be generalized. A nationwide survey would give an overall perception of nursing care in Nepal. There is possibility of information and social desirability bias.

## CONCLUSIONS

The study showed that most of the patients were satisfied with the nursing care provided in a tertiary care centre. Routine nursing care surveys and immediate feedbacks would keep the authorities updated and deliver good health care.

## Conflict of Interest:

None.
